# The impact of long-term mulching cultivation on soil quality, microbial community structure, and fruit quality in “Wanzhou Red Mandarin” citrus orchard

**DOI:** 10.3389/fmicb.2025.1616151

**Published:** 2025-09-04

**Authors:** Yiding Liu, Yanqi Teng, Jie Zheng, Aziz Khan, Xiang Li, Junlin Cui, Krishan K. Verma, Qigao Guo, Kai Zhu

**Affiliations:** ^1^College of Horticulture and Landscape Architecture, Southwest University, Chongqing, China; ^2^Chongqing Three Gorges Vocational College, Chongqing, China; ^3^Institute of Cotton Research, Chinese Academy of Agricultural Sciences, Anyang, China; ^4^College of Agronomy, Shandong Agricultural University, Tai’an, China; ^5^Sugarcane Research Institute, Guangxi Academy of Agricultural Sciences, Nanning, China

**Keywords:** grass mulching, microbial community structure, fruit quality, physicochemical properties of soil, citrus orchards

## Abstract

Mulching, a widely recognized agricultural practice, involves covering the soil surface with organic or inorganic materials to enhance soil properties and optimize growing conditions. This practice has demonstrably positive effects on soil physicochemical and biological properties, leading to reduced evaporation and weed suppression. This study investigated the effects of grass mulching (GV, *Vulpia myuros*) versus clean tillage (CK) on soil properties, microbial communities, and fruit quality in a red mandarin orchard. Grass mulching significantly enhanced the surface soil nutrients (0–20 cm), alkali-hydrolyzable nitrogen (AN, 105.5%), available phosphorus (AP, 144.4%), available potassium (AK, 102.1%), soil organic matter (SOM, 42.5%), total organic carbon (TOC, 93.1%), and enzyme activities, i.e., alkaline phosphatase (60.1%), urease (39.3%), and soil deep layer (20–40 cm) showing lower but notable improvements of available phosphorus (116.6%), total organic carbon (101.9%), respectively. Grass mulching enhanced the *Proteobacteria* abundance (soil surface 36 to 39%, & deep 33 to 37%) and altered fungal dominance (surface: unclassified_*Agaricomycetes*, deep: *Mortierella*). Beta diversity revealed distinct microbial clustering between treatments. Soil physicochemical properties (alkali-hydrolyzable nitrogen, electrical conductivity and soil organic matter) strongly correlated with unclassified_*Micropepsaceae* and *Agaricomycetes*. Grass mulching improved fruit quality, increased vitamin C (24.5%), and decreased pericarp thickness (27.1%), with bacterial communities showing stronger fruit quality correlations than fungi. These results demonstrated that the grass mulching enhances soil fertility, microbial activity and fruit quality, supporting its adoption in sustainable citrus cultivation in the years to come.

## 1 Introduction

The citrus cultivation in China has a long history, and overall planting areas (over 90%) are concentrated in the subtropical climatic regions south of the Yangtze River (Yin et al., 2025). In recent years, China’s cultivated area, production volume, and per capita consumption of citrus fruits have been continuously rising, with remarkable economic values (Zhong et al., 2025; Dong et al., 2024; Zeng et al., 2024). As a significant citrus-producing region in China, Chongqing possesses many citrus resources, including various representative and advanced varieties (Huang et al., 2024). The cultivation history of “Wanzhou Red Mandarin” exceeds 4,000 years. As a characteristic citrus variety of Wanzhou, Chongqing, its planting area is more than 10,000 ha. However, the improper development and growth of citrus orchards may lead to accelerated soil and water loss in the orchards. Soil and water loss significantly affects the soil nutrients and land productivity (Liu et al., 2024). Meanwhile, according to the previous studies, alterations in the soil microbial structure typically occur in orchards with consecutive multi-year cultivation (Wang et al., 2023; Wu et al., 2021). The composition of soil microorganisms is significantly associated with plant growth, yield and fruit quality (Sas-Paszt et al., 2023; Jin et al., 2023).

The physicochemical properties of soil and the biological activities of soil microorganisms collectively influence the ecological processes and functions in the soil ecosystem. A favorable soil environment provides stable environmental conditions for proper plant development (Alori et al., 2022). Consequently, soil protection is a significant component of sustainable agricultural development, with soil coverage as a key protective measure (Koudahe et al., 2022). In the conventional cultivation and management of citrus orchards, clean tillage is the predominant agricultural practice employed. However, prolonged reliance on this method has led to various issues, such as diminished soil fertility within the orchards, reduced biodiversity, compromised fruit quality, and increased physiological diseases affecting the flower and fruit quality (Tu et al., 2021).

Grass mulching refers to the natural growth of grass or the intentional cultivation of annual or perennial herbaceous plants in the inter-row spaces of orchards or throughout the entire orchard, and these plants can be moved or incorporated into the soil as green manure. This cultivation strategy, which is functionally comparable to practices, such as the enhanced application of organic fertilizers, ridge cultivation, optimized row spacing, and plastic film mulching, can significantly improve the physicochemical properties of the soil and enhance the orchard environment (Tang et al., 2022; Yang et al., 2022; Luo et al., 2021). While grass mulching has shown benefits for orchards in China, its adoption has historically been relatively low due to various factors, i.e., regional differences in climatic variables, soil conditions, fruit tree types, and variations in management practices (Wei et al., 2017; Li et al., 2018). This phenomenon can be attributed to specific traditional beliefs held by fruit growers. Many of them perceive that the presence of grass cover may compete with fruit trees for space and soil nutrients. However, the ongoing research suggests that the grass mulching offers numerous benefits, including enhancing soil nutrient content, promoting microbial diversity, suppressing weeds, and improving soil profile (Chen et al., 2014; Licznar-Malanczuk, 2020; Granatstein and Mullinix, 2008). In fruit cultivation, grass mulching has emerged as a significant management strategy and widely promoted in fruit orchards across China.

Most existing research has primarily focused on the effects of citrus orchard covering practices on the physical and chemical properties of the soil. The impact of *Vicia villosa* hirsuta mulching in citrus orchards was compared between clean tillage, four-year mulching, and eight-year mulching treatments, respectively. The research findings revealed that the duration of mulching increased; there was more pronounced enhancement in soil enzyme activities. The enzymatic activities are enhanced (2.45 to 54.42%) compared to other treatments during the eight-year mulching treatment (Wang et al., 2022). Soil properties influenced by arbuscular mycorrhizal fungi, as compared to conventional tillage practices, no-tillage orchards and grass-covered citrus orchards, exhibited significant enhancements in soil organic carbon, available nitrogen content, and soil enzyme activities (Wang et al., 2016; Pang et al., 2022).

Fescue mulching can improve soil characteristics in citrus orchards subjected to continuous cropping systems. It reduces average and maximum soil temperatures while enhancing root exudate yield (Visconti et al., 2022). As essential components of the plant–soil interaction system, soil microorganisms are vital in sustaining soil nutrient cycling and energy flow. With modern biotechnology’s rapid advancement, sequencing costs have steadily decreased (Hemkemeyer et al., 2021). Research on soil microorganisms has become a prominent study area in recent years, with growing recognition of their vital role in ecosystem health and various other fields. The application of mulching treatment increased soil nutrient content and enhanced the richness of arbuscular mycorrhizal fungi (AMF) communities (Fallah et al., 2023); however, it did not lead to significant changes in the diversity of these AMF communities (Xiao et al., 2022).

The intercropping of alfalfa and hairy vetch in citrus orchards has enhanced soil nutrient levels and promoted microbial diversity. Specifically, incorporating alfalfa into these systems may enhance *Penicillium* (58.72%) and *Streptomyces* populations (17.90%), thereby improving the overall soil conditions within citrus orchards (Xie et al., 2024). The study of Gannan navel oranges showed that the mulching treatment significantly enhanced the fungal diversity in soil compared to clean tillage. Additionally, there was a notable enhancement in soil enzyme activities following this treatment (Guan et al., 2024). In studies on other fruit trees, such as apple, peach, persimmon and pear, artificial living grass mulch has enhanced the quantity and activity of beneficial soil microorganisms within orchards (Guo et al., 2024; Yang et al., 2024; Sosna and Fudali, 2023; Suo et al., 2019). This practice improves soil nutrient content and accelerates material cycling, increasing fruit yield and quality. However, few relevant studies have been reported on citrus. Meanwhile, previous studies on the correlation between soil microorganisms, nutrients and fruit quality focused on comparing different mulching varieties. At the same time, there were relatively limited studies on the differences among different soil layers.

In the present study, two treatments, i.e., clean tillage and mulching with *Vulpia myuros* L., were applied in the mandarin orchard in Wanzhou, Chongqing, China. The differences in soil fertility, enzyme activities, and soil microbial communities at different soil depths, i.e., 0–20 and 20–40 cm, were analyzed, and correlation analysis was conducted with fruit quality indicators. Furthermore, we delved deeper into the interaction between plants and the soil environment. The present study was undertaken to evaluate the influence of *V. myuros* cover crops at different soil depths on the soil quality of citrus orchards, assess the effect of *V. myuros* cover on the quality of Wanzhou Red Mandarin, and analyze the correlation between soil quality indicators at different soil depths and the quality of red mandarin. The findings of this research offer theoretical knowledge for the scientific management of mountain grass orchards in the Wanzhou area and the promotion of grass planting in orchards.

## 2 Materials and methods

### 2.1 Location of the study area

The present experiment was demonstrated at the Ancient Hongju (a variety of tangerine) Planting Demonstration Base, operated by the Xinli Vegetable Professional Cooperative in Wanzhou District, Chongqing (108°53′E, 30°90′N; altitude: 824.7 m), China. The site is situated in a subtropical monsoon humid zone characterized by ample sunlight, abundant rainfall, mild temperatures, an extended frost-free period, and minimal occurrences of frost and snow. The annual sunshine duration was 1168.1 h, while yearly radiation reached 114.77 kcal/cm^2^. The average annual temperature was 16.5°C with an average frost-free period of nearly 200 days. Additionally, the region experiences an average annual precipitation of 1,200 mm.

### 2.2 Test materials

The selected orange trees were Wanzhou Red Mandarin, aged 20 years. The row and plant spacing was set at 5 × 5 m. To investigate the effects of grass mulching on soil and fruit quality, two treatments were implemented in 2019. One treatment was associated with clean tillage (CK), while the other consisted of coverage with *Vulpia myuros*. Seeds of *V. myuros* were uniformly sown each year (45 kg/ ha) in May and allowed to grow naturally without mowing. The amounts of total nitrogen, phosphorus pentoxide, and potassium oxide applied were consistent across both treatments according to agricultural management practices. The experiments were designed as a randomized complete block design (RCBD) with three replications.

### 2.3 Soil sampling and determination of physical and chemical properties

The soil samples were collected (July 2024) at five points randomly selected in an “S” shape in each experimental plot from the 0–20 and 20–40 cm soil layers. The samples were subsequently passed through a 2 mm sieve and thoroughly mixed, resulting in 12 individual soil samples (*n* = 12). Each soil sample was divided into three parts and placed in self-sealing bags in a sample box with ice packs for low-temperature transport to the laboratory. After the samples were brought to the laboratory, one part of the samples was used for the analysis of physicochemical properties of the soil, and the other part was stored at 4°C in a refrigerator for subsequent analysis of soil enzyme activities, microbial biomass, carbon and nitrogen, as well as available nutrient content. The third sub-sample was stored at −80°C in a deep freezer for the microbial community structure analysis.

Soil organic matter (SOM) was determined by the K_2_CrO_7_-H_2_SO_4_ oxidation method (Schulte and Hoskins, 1995). The Kjeldahl method determined soil total nitrogen (TN) and alkaline-hydrolyzable N (AN). Available phosphorus (AP) and total phosphorus (TP) were determined by the molybdenum-antimony-antipyrine colorimetric method. The available potassium (AK) was extracted with NH_4_OAc and quantified by flame photometry. Microbial biomass nitrogen (MBN) was analyzed by the chloroform fumigation-extraction method. The pH in the soil was measured in a 1:2.5 soil/ water mixture using a digital pH meter (Starter-2100 pH) (Wilke et al., 2005). The total organic carbon (TOC) content was analyzed by the dichromate titration method with Toc-ssm-5000A (Shimadzu, Japan) (Bao, 2000). Soil urease (URE) and sucrase (SUA) activities were determined by the sodium phenolate colorimetric and 3,5-dinitrosalicylic acid colorimetric methods. Alkaline phosphatase (ALP) and soil catalase (CAT) content were determined by the sodium phenyl phosphate colorimetric and ultraviolet spectrophotometry methods (Zhou and Zhang, 1980; Yang et al., 2011).

### 2.4 High-throughput sequencing of soil microorganisms

Total genomic DNA was extracted from the soil samples by TGuide S96 Magnetic Soil /Stool DNA Kit (Tiangen Biotech Co., Ltd., Beijing, China) according to the manufacturer’s instructions. The hypervariable region V3-V4 of the bacterial 16S rRNA gene was amplified with primer pairs, such as 338F: 5′- ACTCCTACGGGAGGCAGCA-3′ and 806R: 5′- GGACTACHVGGGTWTCTAAT-3′. PCR products were checked on agarose gel and purified through the Omega DNA purification kit (Omega Inc., Norcross, GA, USA). The purified PCR products were collected, and paired ends (2 × 250 bp) were performed on the Illumina Novaseq 6,000 platform. The Internal Transcribed Spacer (ITS) variable regions of fungi were amplified using the ITS1FI2 (5’-GTGARTCATCGAATCTTTG-3′) and ITS2 (5’-TCCTCCGCTTATTGATATGC-3′) primers. The ITS region, located between the small subunit (SSU) and large subunit (LSU) ribosomal RNA genes, is highly variable and commonly used for fungal identification and phylogenetic analysis. The amplification procedure and conditions were the same for the 16S rDNA. The amplified products were subjected to high-throughput sequencing using the Illumina HiSeq 2,500 PE250 second-generation sequencing platform. The Beijing Biomarker Biotechnology Co., Ltd., China, provided the sequencing service.

### 2.5 Bioinformatic analysis

The qualified sequences with more than 97% similarity thresholds were allocated to one operational taxonomic unit (OTU) using USEARCH (version 10.0). Taxonomy annotation of the OTUs/ASVs was performed based on the Naive Bayes classifier in QIIME2 (Hall and Beiko, 2018) using the SILVA database (Quast et al., 2012) (release 138.1) with a confidence threshold of 70%. Alpha diversity was performed to identify each sample’s species diversity complexity utilizing QIIME2 software. Beta diversity calculations were analyzed using principal coordinate analysis (PCoA) to assess the diversity of species complexity. One-way analysis of variance (ANOVA) was used to compare bacterial abundance and diversity. Linear discriminant analysis (LDA) coupled with effect size (LEfSe) was applied to assess the differentially abundant taxa. Clean RNA-seq data were obtained, and bioinformatics analysis was performed using the *BMKCloud platform*^1^.

### 2.6 Physiological index measurement

Single fruit weight (SFW), fruit skin thickness (FT), longitudinal diameter (LD) and transverse diameter (TD) were monitored with a vernier caliper. Single fruit weight was measured with a digital balance. Fruit soluble solid content was observed to peel off the fruit skin, take 3 to 5 sections from different positions of the fruit, grind the pulp into juice, and measure with a handheld sugar-acid meter. Zero the meter and rinse it three times before each measurement. Perform parallel tests thrice and calculate the average value. Vitamin C content was observed using the 2,6-dichlorophenol titration method.

Fruit shape index (FSI) was calculated as:

Fruit shape index = Transverse diameter / Longitudinal diameter

## 3 Results

### 3.1 The impacts of mulching treatment and different soil layer depths on the physicochemical properties of soil

In the surface soil layer (0–20 cm), the growing *Vulpia myuros* (GV) treatment significantly improved the key soil properties compared to clean tillage (CK). Specifically, AN increased by 105.5%, AP (144.4%), EC (95.6%), SOM (42.5%), AK (102.1%), TN (12.7%), TOC (93.1%), and MBN (10.7%), respectively. In the deep soil layer (20–40 cm), the values increased by 89.3, 116.6, 141.8, 41.2, 32.9, 9.7, and 101.9%, except for MBN activity (−4.3%). The growing *V. myuros* treatment was significantly better than CK in the nutrient indicators, such as AN, AP, AK, organic matter (SOM, TOC), and electrical conductivity (EC) throughout the soil layer. However, the enhancement of pH, TN, and MBN values was only manifested in the surface soil (0–20 cm). Regardless of the specific treatment approaches, it is generally observed that the surface soil (0–20 cm) contains significantly higher levels of nutrients, organic matter, and microbial biomass nitrogen compared to the deep soil layer (20–40 cm). This is a common trend in soil profiles, with the surface layer typically exhibiting greater biological activity and higher concentrations of these key soil components (Table 1).

**Table 1:** Soil physical and chemical indices in different soil layers (0–20 cm and 20–40 cm) under different treatments (GV, growing *Vulpia myuros*; CK, clean tillage).

Indicator	CK		GV	
	0–20 cm	20–40 cm	0–20 cm	20–40 cm
pH	5.53 ± 0.04^b^	5.31 ± 0.04^c^	5.79 ± 0.03^a^	5.25 ± 0.05^c^
AN (mg/kg)	70.04 ± 2.92^c^	60.53 ± 2.98^d^	143.92 ± 5.38^a^	114.63 ± 5.59^b^
AP (mg/kg)	30.58 ± 0.05^b^	11.69 ± 0.18^c^	74.75 ± 0.31^a^	25.32 ± 0.23^b^
EC (μS/cm)	81.63 ± 0.85^c^	51.07 ± 0.40^d^	159.73 ± 4.57^a^	123.53 ± 3.21^b^
SOM (g/kg)	14.23 ± 0.43^b^	11.54 ± 0.56^c^	20.28 ± 0.12^a^	16.3 ± 0.08^b^
AK (mg/kg)	75.13 ± 0.31^b^	63.1 ± 0.19^c^	151.8 ± 0.63^a^	83.87 ± 0.41^b^
TN (g/kg)	1.34 ± 0.03^ab^	1.03 ± 0.05^c^	1.51 ± 0.04^a^	1.13 ± 0.02^c^
TOC (g/kg)	15.11 ± 0.32^b^	8.38 ± 0.31^c^	29.18 ± 0.48^a^	16.92 ± 0.25^b^
MBN (mg/kg)	25.2 ± 0.28^a^	17.99 ± 0.51^b^	27.89 ± 0.25^a^	17.22 ± 0.45^b^

Different lowercase letters in the table denote significant differences (*p* < 0.05 by LSD test) among the treatments. The mean ± SD is presented under different treatments. AN, alkali-hydrolyzable nitrogen; AP, available phosphorus; EC, electrical conductivity; SOM, soil organic matter; AK, available potassium; TN, total nitrogen; TOC, total organic carbon; MBN, microbial biomass nitrogen.

### 3.2 Analysis of soil enzyme activities under different treatments

**Figure 1:**
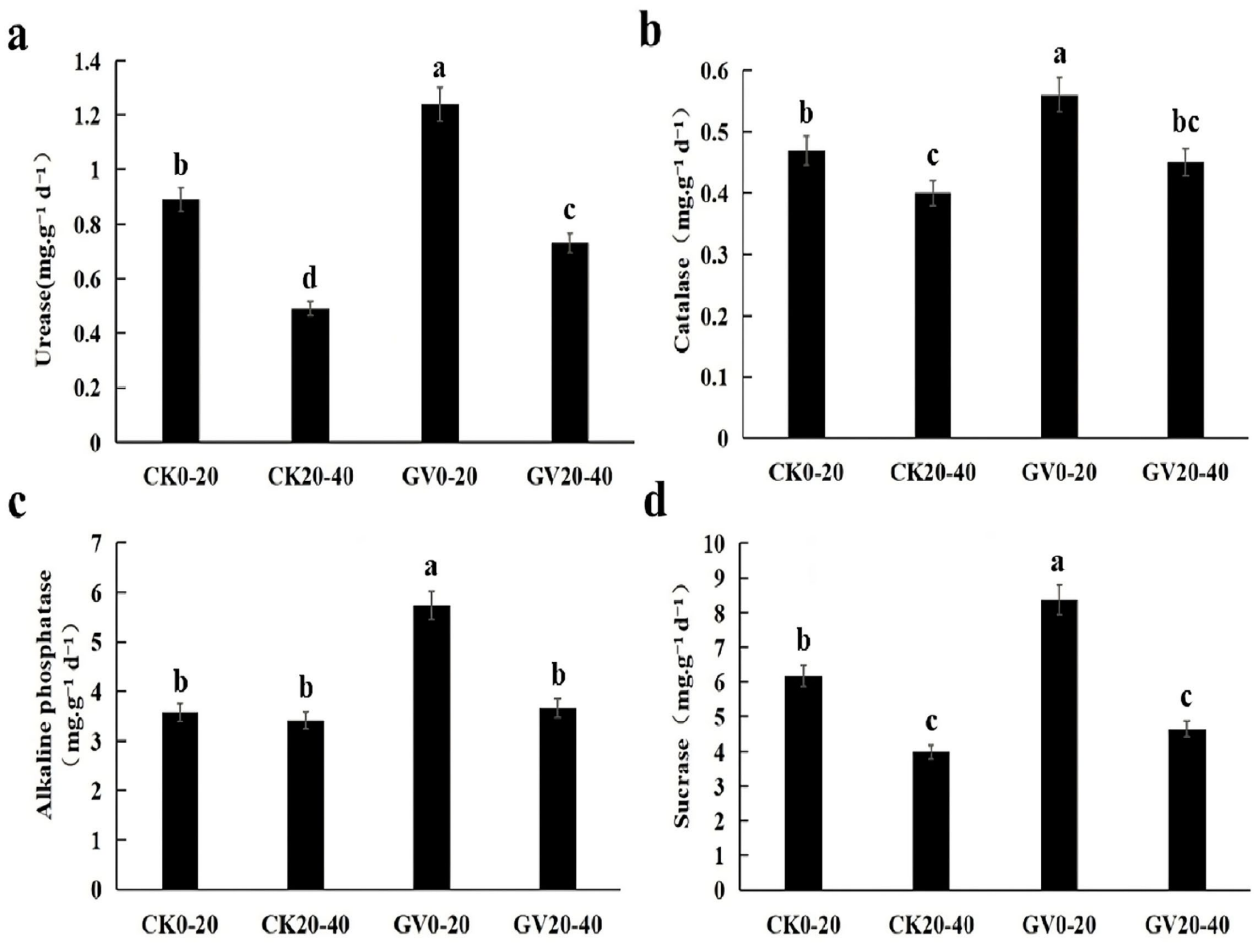
The influences of different treatments on soil enzyme activities in the 0–40 cm soil layer of red mandarin orchards in Wanzhou, Chongqing **(a)** The differences in urease activities among the four samples; **(b)** The differences in catalase activities among the four samples; **(c)** The differences in alkaline phosphatase activities among the four samples; **(d)** The differences in sucrase activities among the four samples. Different lowercase letters indicate the significant differences (*p < 0.05 by LSD) among other treatments.

In the GV treatment, all enzyme activities in the deep soil (20–40 cm) were significantly lower than in the surface layer (0–20 cm). The activities of sucrase (−44.4%) and urease (−41.1%) were downregulated most conspicuously. The alkaline phosphatase activity in the deep soil remained 63.9% of the surface layer. The activity of CAT decreased the least, amounting to −19.6%. In the CK treatment, the activities of urease and sucrase in the deep soil were reduced significantly (−44.9 and −35.6%), which was in line with organic matter accumulation in the surface layer; the alkaline phosphatase activity in the deep soil under CK treatment was downregulated by nearly 4.7%. When comparing the same soil layers between the GV and CK treatments, in the 0–20 cm soil layer, the GV treatment significantly enhanced the activities of all enzymes, with alkaline phosphatase (+60.1%) and urease (+39.3%) being the most prominent. In the 20–40 cm soil layer, the GV treatment enhancement of deep soil enzyme activities was generally lower than that of the surface layer. Still, urease activity increased significantly (+48.9%) as shown in Figure 1.

### 3.3 The impacts of diverse tillage practices on soil microbial communities

**Figure 2:**
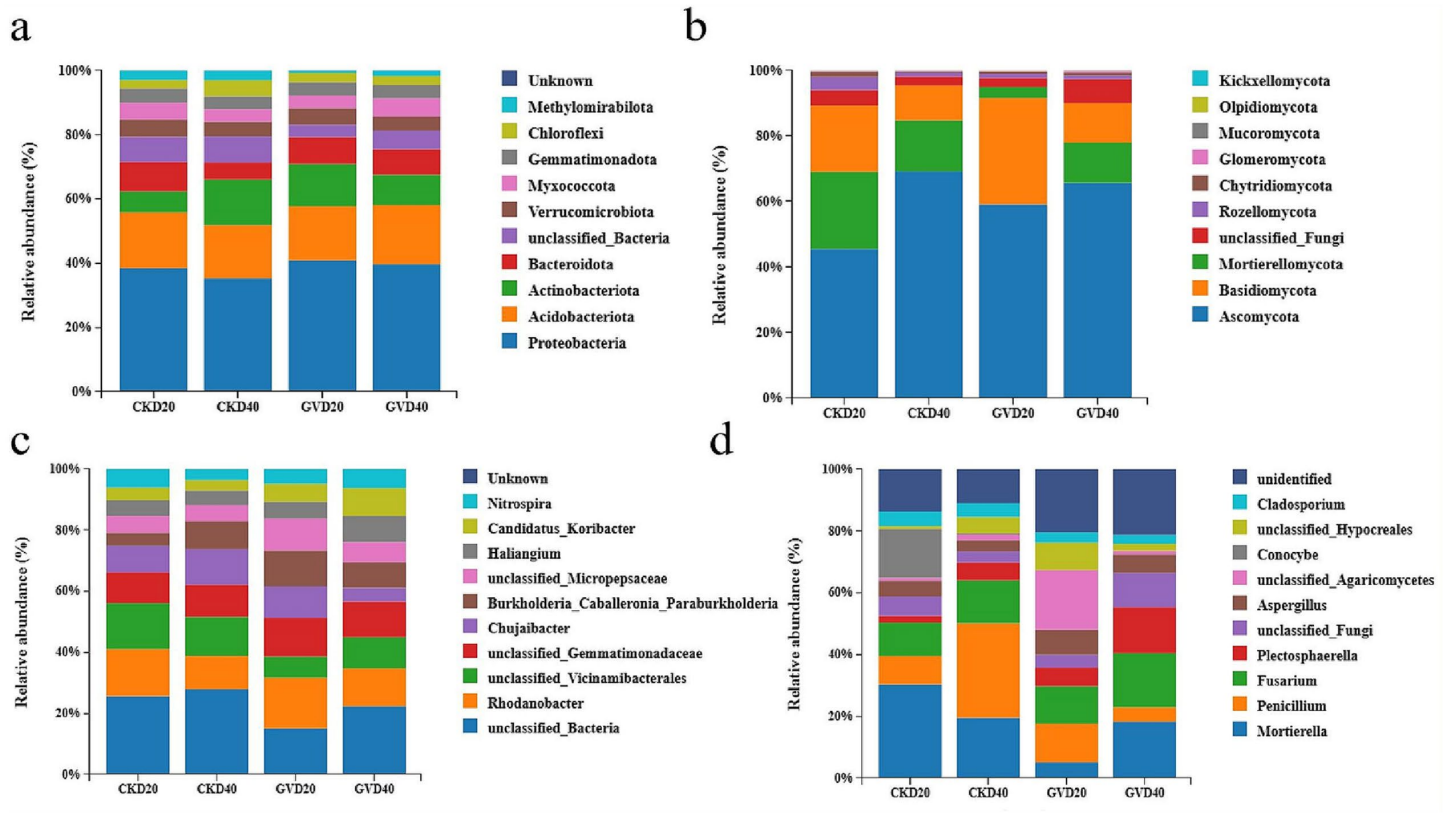
The relative abundances of soil bacterial and fungal communities at different soil layers (0–20 cm, 20–40 cm) under two tillage practices, ranging from phylum **(a,b)** to genus **(c,d)**. The abscissa is the sample names CK (conventional tillage) and GV (growing *Vulpia myuros*), and the ordinate is the relative abundance.

Based on the comprehensive assessment of the soil bacterial communities under diverse treatments, the five most abundant phyla were observed, such as *Proteobacteria*, *Acidobacteriota*, *Actinobacteriota*, *Bacteroidota*, and unclassified_*Bacteria*, accounting for 73.42–78.74% of the total bacterial abundance in the soil of the red mandarin orchard. *Proteobacteria* and *Acidobacteriota* were the most predominant bacterial phyla in the soil, constituting 32.78–38.69% and 15.36–17.28%, respectively. The vertical distribution of *Proteobacteria* manifested in all treatments, and its relative abundance declined with the increase of soil depth. Meanwhile, the mulching treatment significantly upregulated the relative abundance of *Proteobacteria* at all soil depths (GVD20 enhanced from 35.56–38.69% compared with CKD20, and GVD40 rose from 32.78–37.26% compared with CKD40). The relative abundance of *Acidobacteriota* was relatively stable, with no significant discrepancies among the groups. Nevertheless, the distribution pattern of *Actinobacteria* was contrary to that of *Proteobacteria*. Its relative abundance in the surface soil (0–20 cm) was lower than in the deep soil (20–40 cm). Under the mulching approach, its relative abundance in the 0–20 cm soil layer escalated in the CKD20 (6.06%), while in the 20–40 cm soil layer (12.40%), it decreased from 13.17% in CKD40 to 8.80% (Figure 2a). In the comparison at the bacterial genus level, the mulching treatment downregulated the relative abundances of unclassified_*Bacteria* and unclassified_*Vicinamibacterales* while enhancing the relative abundances of *Rhodanobacter* and unclassified_*Micropepsaceae* (Figure 2c).

The analytical results of the fungal community showed that the top five in terms of relative abundance were observed, such as *Ascomycota*, *Mortierellomycota*, *Basidiomycota*, unclassified-*Fungi,* and *Rozellomycota* among the GV and CK cultivation measures on all soil layer depths. Among all bacterial phyla, they account for 98.01 to 99.38%. In all treatments, the *Ascomycota* was the most predominant phylum, with values ranging from 45.55 to 69.30%, and its abundance was even higher in the deep soil layers (CKD40, GVD40). Under the clean tillage treatment, the abundance of *Ascomycota* in the 0–20 cm soil layer increased (45.55%) in CKD20 and CKD40 (69.30%). However, under the treatment of covering with *Vulpia myuros*, it enhanced 59.21% in GVD20 and 65.77% in GVD40. The GV treatment augmented the abundance of *Ascomycota* in the 0–20 cm soil layer, thereby significantly reducing the extent of its increase with soil depth (Figure 2b). The community composition differed substantially among various treatments at the fungal genus level analysis. In the CK treatment, the vertical distribution along the soil layer presented that the surface soil (0–20 cm) was dominated by *Mortierella* (with a relative abundance of 23.34%), and in the deep layer (20-40 cm), *Penicillium* became dominant (increasing from 7% in CKD20 to a relative abundance of 23.86%). In the GV treatment, unclassified_*Agaricomycetes* had the highest relative abundance in the surface soil (12.82%), which was not detected in other treatments. In the deep soil, *Mortierella* resumed dominance (increasing from 3.30% in GVD20 to relative abundance of 12.17%), but *Penicillium* was significantly suppressed compared to CKD40 (23.86%) and GVD20 (8.30%), with relative abundance of only 3.10% (Figure 2d).

**Figure 3:**
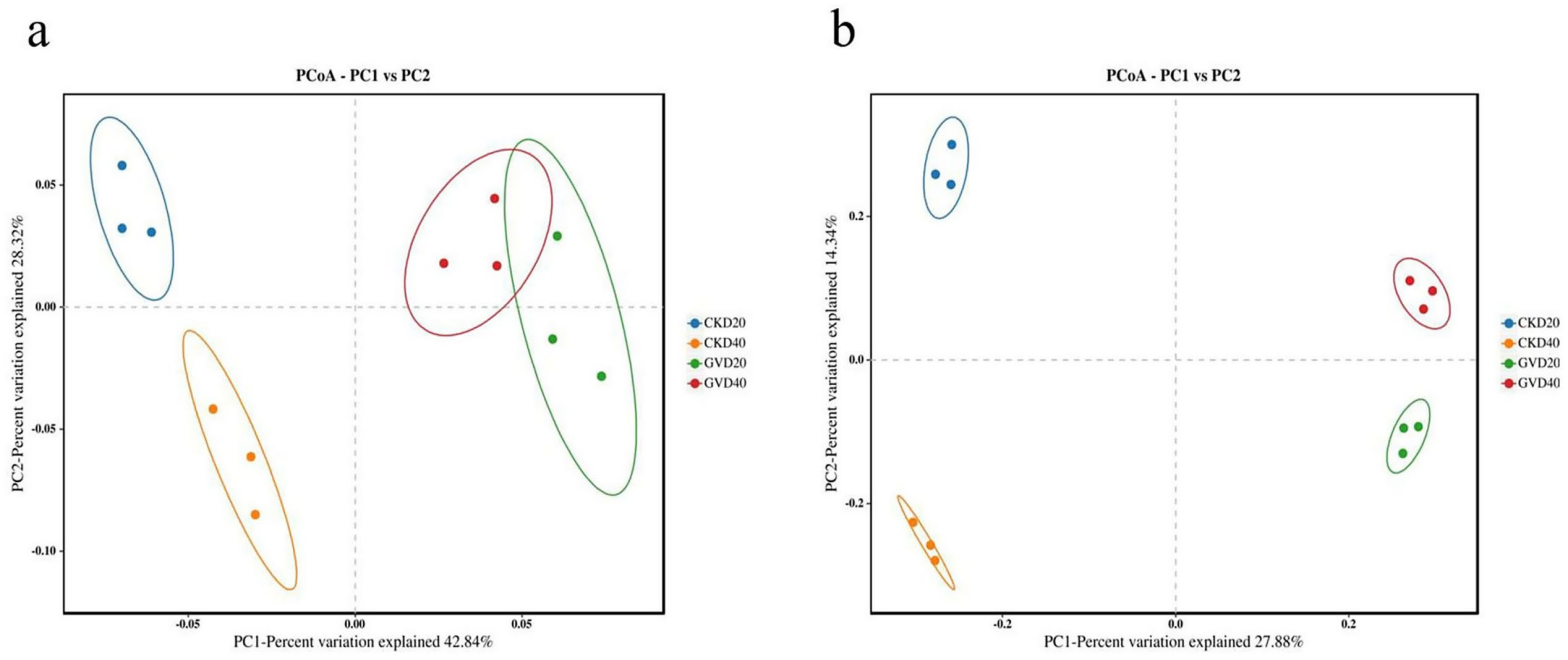
Principal coordinate analysis (PCoA) of soil microbial communities at various soil depths (0–40 cm) under clean tillage and grass mulching, **(a)** bacteria, **(b)** fungi, CKD (clean tillage), GV (growing *Vulpia myuros*).

Beta diversity analysis was performed using principal coordinate analysis (PCoA) to compare the extent of similarity in species diversity among different samples. The results demonstrated that the bacterial communities among the various groups under the mulching and clean tillage measures could be categorized into three clusters. There were notable dissimilarities between CKD20 and CKD40, while the distinctions between GVD20 and GVD40 were not conspicuous. Additionally, the disparity between GV and CK was relatively substantial. In the PCoA analysis of the fungal communities, marked differences were observed among different treatments and soil layers, which were aggregated into four types. These outcomes signified that the mulching treatment (GV) significantly modified the soil’s microbial communities, encompassing bacteria and fungi (Figure 3).

### 3.4 Correlation analysis between soil physicochemical properties and microbial community structure

**Figure 4:**
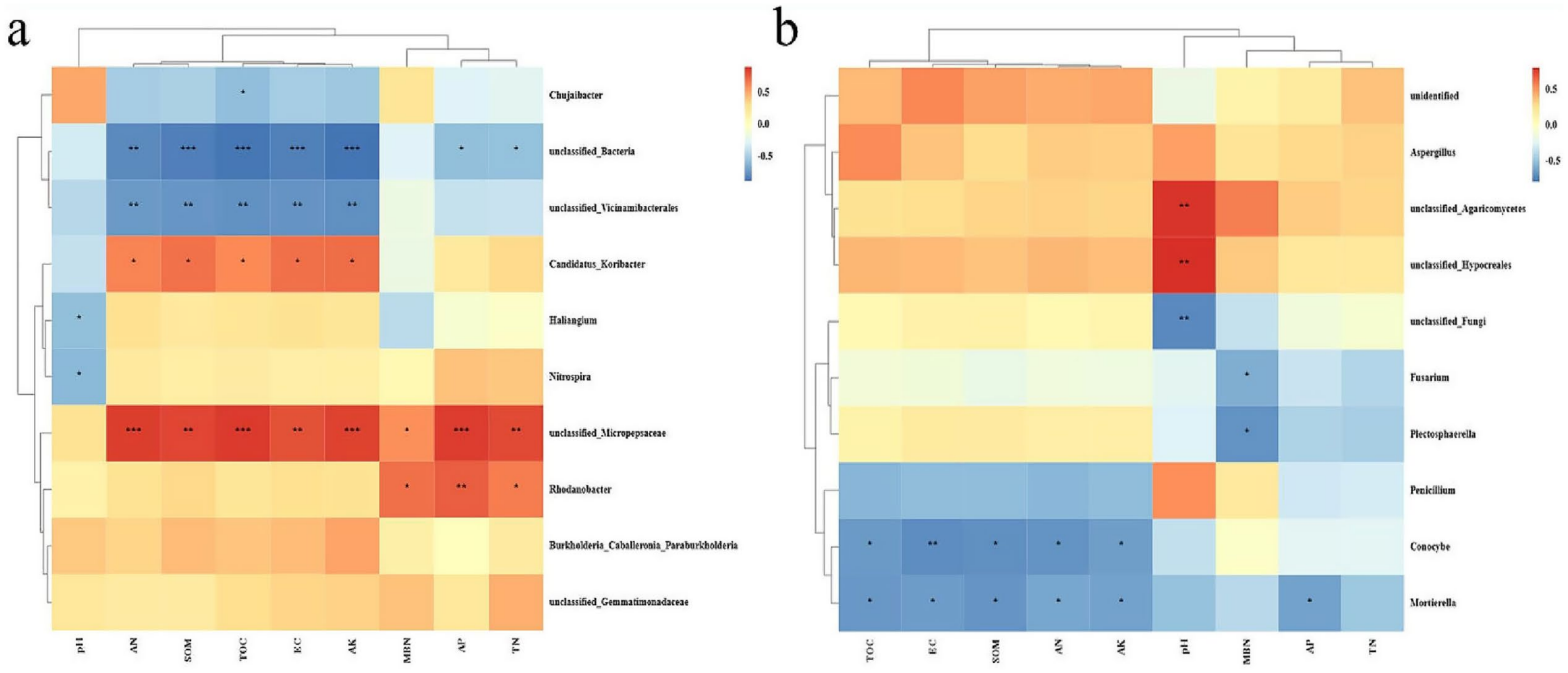
Heatmap for the correlation analysis of soil physicochemical property indicators and soil microbial community structure, **(a)** bacteria, **(b)** fungi (at the genus level) based on Pearson analysis. The red color indicates positive correlation and blue indicates negative correlation, and the deeper the color, the stronger the correlation. The symbols in the figure indicate the degree of significance (*p < 0.05; **p < 0.01; *** *p* < 0.001).

Based on the Pearson analysis, correlation analysis was conducted on the top 10 bacteria and fungi at the genus level, and the contents of soil properties, i.e., pH, AN, SOM, TOC, EC, AK, MBN, AP, and TN, to assess the relationship between soil physicochemical properties and microbial community structure. The results indicated that the unclassified_*Micropepsaceae* exhibited significant positive correlation with all indicators except pH and MBN among bacteria. At the same time, unclassified_*Bacteria* presented an extremely substantial negative correlation with SOC, TOC, EC, and AK, and the genus *Rhodanobacter,* and AP have significant positive correlation. Among fungi, *Conocybe* and *Mortierella* were negatively correlated with most soil indicators. Specifically, the unique unclassified_*Agaricomycetes* in GVD20 had significant positive correlation with pH. Among the soil indicators, AN, SOM, TOC, EC, and AK had a stronger correlation with the top 10 bacteria, while pH had a stronger correlation with fungi (Figure 4).

### 3.5 The impact of covering measures on the fruit quality of red mandarin

The results of the fruit quality indicators revealed the significant disparity in the fruit quality of Wanzhou Red mandarins between the GV and CK treatments. Among all the tested indicators, except the pericarp thickness (PT) under the grass mulching treatment, which significantly decreased compared to clean tillage measure (27.10%), others showed significantly upregulating trends. Among them, the vitamin C content (VC) had the most significant increase (24.5%), while the transverse diameter (TD) had the lowest enhancement (6.76%). Meanwhile, the soluble solids (SS) content increased by 10.90%. It suggests that the grass cultivation can promote the growth of Wanzhou red mandarin fruits and the synthesis of vitamin C content (Table 2).

**Table 2:** The impact of different measures on the fruit quality of Wanzhou Red Mandarin.

Treatment	SFW	LD	TD	FSI	PT	SS	VC
CK	17.03 ± 0.09^b^	5.77 ± 0.19^b^	7.24 ± 0.26^b^	0.80 ± 0.05^b^	4.22 ± 0.70^b^	10.55 ± 0.20^b^	36.7 ± 1.20^b^
GV	19.91 ± 0.25^a^	6.89 ± 0.61^a^	7.73 ± 0.20^a^	0.89 ± 0.07^a^	3.32 ± 0.55^a^	11.70 ± 0.60^a^	45.7 ± 1.30^a^

The differences under the treatments of clean tillage (CK) and cover with Vulpia myuros (GV), SFW, single fruit weight (g); LD, longitudinal diameter (cm); TD, transverse diameter (cm); FSI, fruit shape index; PT, pericarp thickness (mm); SS, soluble solids content (%); VC, vitamin C content (mg/100 g). Different letters denotes significant differences among different treatments based on the LSD test (*p* < 0.05).

**Figure 5:**
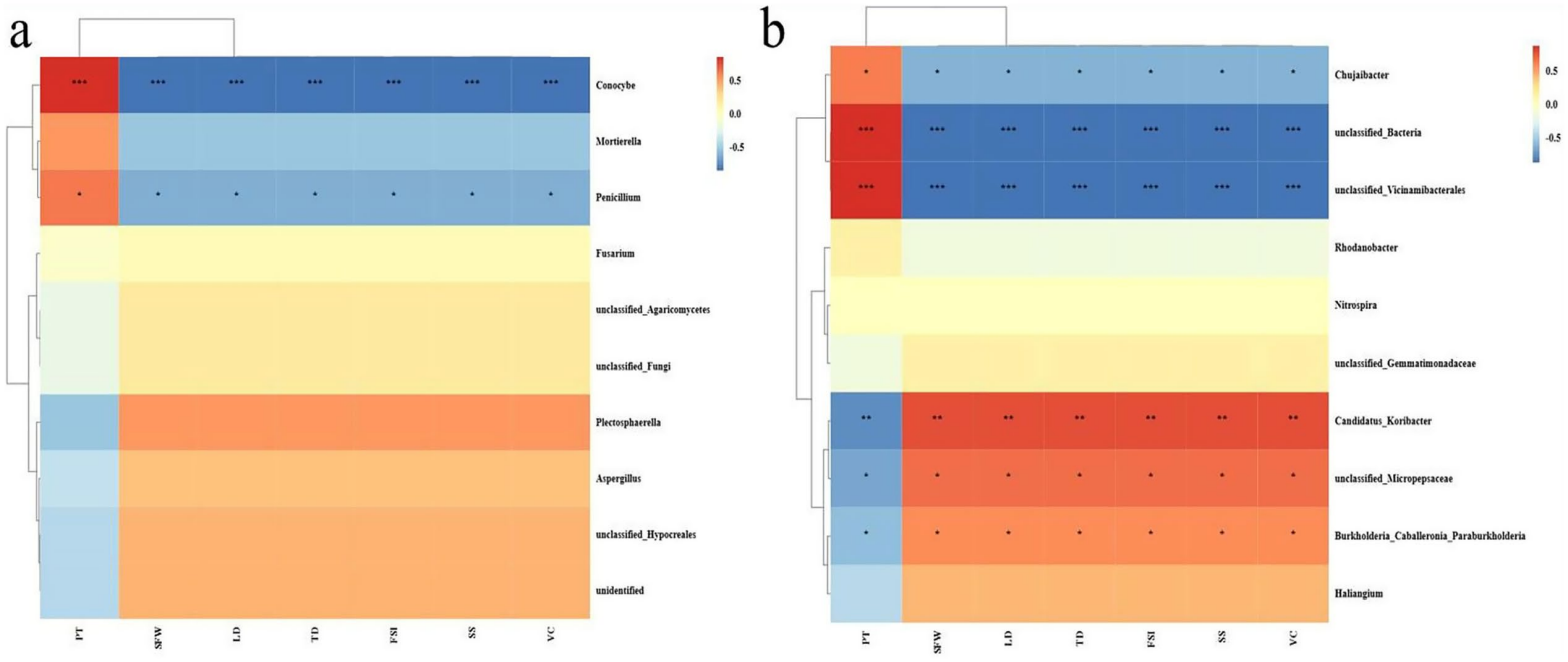
Heatmap depicting the interrelationships between soil microorganisms and fruit quality indicators under mulching and clean tillage measures based on Pearson analysis, where **(a)** represents fungi, and **(b)** represents bacteria. The red color indicates positive correlation and blue indicates negative correlation, and the deeper the color, the stronger the correlation. The symbols in the figure indicate the significance level (*p < 0.05; **p < 0.01; ***p < 0.001).

A correlation analysis was conducted between the fruit quality indicators and the soil microbial community structures at the genus level using different distinct tillage measures. The results indicated that more bacteria than fungi exhibited significant correlations with the fruit quality indicators. Specifically, among the fungi, only *Conocybe* and *Penicillium* presented substantial negative correlations with the fruit quality indicators, excluding FT. In contrast, within the bacteria, six species, such as *Chujiaibacter*, *Candidatus-Koribacter*, unclassified-*Bacteria*, *Vicinamibacterales*, unclassified-*Micropepsaceae*, and *Burkholderia-Caballeronia-Paraburkholderia*, showed significant positive or negative correlations with fruit quality indicators (Figure 5).

## 4 Discussion

### 4.1 The impact of mulching treatment on soil physicochemical properties

The content and composition of soil organic matter are intricately associated with the quality and fertility of soil. It serves as a crucial foundation for sustaining the stability and promoting the sustainable development of the soil ecosystem (Liang et al., 2017). The genetic factors may influence the role of soil microorganisms. Genetic phenomena, i.e., horizontal gene transfer, which is associated with the exchange of genetic material between microorganisms, can introduce advanced traits and impact community dynamics (Kiprotich et al., 2025). In this study, the grass-covering measure (GV) significantly enhanced the organic matter content in 0–20 cm soil depth, with an enhancement of 42.5% compared to the clean tillage treatment. The improvement in SOM and TOC due to the GV treatment was diminished in 20–40 cm soil depth. However, it remained significantly higher than observed in the control group. Specifically, SOM increased from 11.54 to 16.3 g/kg, while TOC rose from 8.38 to 16.92 g/kg. This phenomenon is associated with the input of organic carbon, such as plant root litter under the GV treatment, and the surface soil accumulates organic matter more significantly because of sufficient oxygen and high microbial activities (Shi et al., 2018).

Deep soil organic matter accumulates primarily through the gradual decomposition of surface-derived organic matter and leaching of soluble compounds. However, this process occurs much more slowly than topsoil, resulting in a more limited enhancement in the organic matter content. Leaching moves soluble organic compounds deeper into the soil profile, while microbial decomposition breaks organic material into simpler forms. Recent studies have identified a significant increase in TOC in subsurface soil layers (20–40 cm), which appears to be strongly associated with the vertical migration of small-molecule soluble organic carbon. This phenomenon has important implications for carbon sequestration and soil health (Jobbágy and Jackson, 2000).

Inorganic nutrients in the soil are indispensable for supporting the normal growth and development of plants. These nutrients are key components absorbed from the rhizospheric soil to facilitate essential biological processes. Among these nutrients, nitrogen (N), phosphorus (P), and potassium (K) are the primary macronutrients due to the significant quantities required for the plant physiological activities (Jones and Jacobsen, 2005). Numerous previous studies have demonstrated that grass cultivation may significantly improve the essential nutrients in the soil. For instance, this has been observed in loquat, apple and pear plants (Duan et al., 2020; Cao et al., 2021; Yu et al., 2024). In the research on the citrus variety “Taoye Cheng,” a comparison between ryegrass mulching and clean tillage revealed increases in the contents of N (31.1%), P (61.1%), and K (47.3%), respectively (Pan et al., 2024). These findings align with this study. However, it is noteworthy that while the increase observed in the present study was more pronounced, the absolute gains were lower. This discrepancy can be attributed to this particular citrus orchard’s relatively lower soil nutrient content. The pH value of soil is considered a fundamental variable that significantly influences the soil physicochemical and biological properties, thereby affecting plant nutrient absorption efficiency (Neina, 2019). The GV treatment increased the soil’s pH from 0 to 20 cm. Conversely, no significant differences were observed in CK and GV treatments at 20–40 cm soil depth. This finding is consistent with the study conducted by Visconti et al. (2024).

### 4.2 The impact of mulching on soil enzyme activities and soil microbial structure

Soil enzyme activities are a vital parameter for assessing soil fertility. It represents one of the most dynamic components in the soil ecosystem as a biological catalyst that facilitates various biochemical processes occurring in the soil (Tabatabai, 1994; Burns et al., 2013). The activity of urease demonstrated significant vertical distribution discrepancies. In both treatments, the enzyme activity in the 20–40 cm soil layer was markedly lower than in the 0–20 cm soil layer. Furthermore, the GV treatment notably enhanced urease activity throughout the entire soil profile, aligning with the findings reported by Li et al. (2022). In addition, the activities of sucrase and catalase in both soil layers under the GV treatment were higher than those under the CK treatment (Xiang et al., 2023; Shi et al., 2024). It can be attributed to the continuous input of active carbon and nitrogen derived from plant residues, such as root exudates and litter, under the cultivation of *Vulpia myuros*. These abundant carbon sources provide sufficient nutrition and ameliorate the soil hydrothermal conditions and pH value, promoting vigorous microbial metabolism and enhancing soil microbial activity and offering a suitable environment for enzymes to survive (Qian et al., 2014).

As a significant source of soil enzymes, soil microorganisms play a crucial role in the metabolism and cycling of soil nutrients. Their communities’ structure directly affects the energy flow rate within the soil ecosystem. Furthermore, microbial diversity is essential for maintaining ecosystem stability and fulfilling various microbial functions. Thus, it is often used as a critical indicator to assess soil nutrient transformation activity and cycle intensity (Foster, 1988). Under the GV treatment, the community structure and abundance of rhizosphere soil microorganisms associated with Wanzhou Red Mandarin were higher than those in clean cultivation. Notably, significant differences were observed in the community composition of bacterial genera at 0–20 cm soil depth, as well as in the community composition of fungal phyla and genera at 0–20 and 20–40 cm soil depths, which is analogous to the consistent changes in microbial community structure with soil depth following the mulching treatment in the pear orchard (Fu et al., 2023). In the bacterial phylum classification level comparison, the most enriched groups under clean tillage and mulching practices were *Proteobacteria*, *Acidobacteriota*, and *Actinobacteriota*. *Proteobacteria* play a significant role in carbon, nitrogen, and sulfur cycles (Spain et al., 2009).

In this study, the predominant genera within this phylum were *Burkholderia* and *Nitrospira*, which are closely associated with organic matter decomposition, nitrification, and denitrification (Salles et al., 2004; Daims et al., 2015). The abundance of *Burkholderia* in the 0–20 and 20–40 cm soil layers under GV treatment increased by 135.0 and 56.6%, respectively; similarly, *Nitrospira* showed increases of 105.5 and 89.3%, respectively. This enhancement may be attributed to the rise in related microorganisms that facilitated organic matter mineralization and nitrification. *Actinobacteria* are known to drive the decomposition of recalcitrant carbon compounds, such as cellulose and chitin (Dai et al., 2018), compared with CK. The relative abundance of *Actinobacteria* in the 0–20 cm soil depth under GV treatment significantly increased by 104%, which was positively correlated with an accumulation of soil organic matter that enhanced up to 42.5% as well as total organic carbon that increased by 93.1% in this surface layer. Conversely, their abundance in the 20–40 cm soil layer decreased by 33%. This decline may be due to insufficient carbon sources limiting microbial activity at greater depths, while adequate carbon availability from mulching provided sufficient energy for microbial processes near the surface. Furthermore, unclassified-*Micropesacea*e exhibited significant positive correlation with soil factors except pH value in the correlation analysis with soil physicochemical properties (Figure 4).

The composition of the fungal community has experienced significant changes compared to soil bacteria, especially in the classification at the genus level. In the comparison at the phylum level, the relative abundances of *Ascomycota* and *Basidiomycota* in the 0–20 cm soil layer treated with GV were significantly enhanced by 30.10 and 60.23%, respectively. However, the relative abundance of *Mortierellomycota* was downregulated (23.51 to 3.38%). Ylänne et al. (2021) discovered that the *Mortierellomycota* was negatively correlated with soil carbon storage, which might stem from its limited capacity to decompose complex organic substances. In the present research, it was negatively correlated with all soil physicochemical indicators, not merely soil carbon storage. This might be associated with the changes in carbon source preference due to the input of high-lignin organic matter from GV. *Mortierella* is often regarded as capable of surviving in extreme environmental conditions and utilizing carbon sources derived from cellulose, hemicellulose, and chitin (Ozimek and Hanaka, 2020). The abundance of unclassified *Agaricomycetes* significantly increased in the soil associated with healthy plant roots (Yang et al., 2023). An intriguing phenomenon was observed under the GV treatment, the relative abundance of *Mortierella* decreased significantly (89.4%) in the 0–20 cm soil depth.

In contrast, the relative abundance of *Basidiomycota* increased by 60%. Notably, unclassified *Agaricomycetes* exhibited a remarkable increase of 17.08% compared to CK. This change may be attributed to *Vulpia myuros* enhancing survival conditions for soil microorganisms. Furthermore, competitive inhibition may occur between unclassified *Agaricomycetes* and *Basidiomycota*, which could alter the composition of the fungal community. In contrast to the 0–20 cm soil depth, no significant changes were observed at the fungal phylum level in the 20–40 cm soil layer under GV treatment. However, compared with CK, both treatments enhanced the fungal diversity.

### 4.3 The impact of mulching cultivation on fruit yield and quality

Orchard grassing, recognized as a sustainable and effective soil management practice in orchards, has been identified as a significant strategy for enhancing fruit yield and quality. Numerous studies have demonstrated that this practice can improve the physical and chemical properties of the soil while increasing the population of beneficial microorganisms (Tworkoski and Glenn, 2010; Yan et al., 2011; Yu et al., 2025). These changes contribute to improved microenvironment within the orchard, thereby promoting the growth of fruit trees and ultimately enhancing the quality and yield of fruits (Ren et al., 2023). Orchard grassing can potentially reduce soil nutrient loss and enhance soil fertility. Previous research indicates that mulching cultivation can significantly improve fruit shape index, vitamin C content, and soluble solids by modifying soil nutrient composition (Li, 2013; He et al., 2010). This practice enhances the esthetic quality of fruits and positively affects various indicators related to individual fruit quality and yield.

Furthermore, mulching cultivation can affect the soil microbial community by decomposing crop residues and root exudates, thereby improving fruit quality (Li, 2021). This study used Pearson’s correlation coefficient to analyze the correlation between fruit quality and soil physicochemical properties. The results indicated a significant positive correlation between total organic carbon and vitamin C content (*r* = 0.93) and a significant positive correlation between ammonium nitrogen and total dissolved solids (*r* = 0.82). These findings may be attributed to enhancing antioxidant substance synthesis in fruits due to improvements in organic matter and promoting cell expansion facilitated by nitrogen supply. Conversely, available phosphorus exhibited a significant negative correlation with the pH value of soil saturation extract (*r* = −0.88), which aligns with the research findings on pomelo plants (Li et al., 2022).

When analyzing the correlation between soil physical and chemical properties and soil microorganisms, it’s common to categorize the soil indicators into two main groups, i.e., physical/chemical and biological. This distinction helps understand the complex relationships and interactions within the soil ecosystem. Notably, the pH value exhibits a contrasting pattern compared to other indicators. We identified a variety of microorganisms that showed significant correlations with the soil’s physical and chemical properties. For instance, among fungi, *Conocybe* and *Penicillium* are significantly negatively correlated with soil nutrients. In contrast, some specific bacteria, such as *Burkholderia-Caballeronia-Paraburkholderia* and *Candidatus-Koribacter*, demonstrated the significant positive correlation with these properties, while *Chujaibacter* reveals the significant negative correlation (Figure 5). These microorganisms might play essential roles in alterations in the microbial community structure and variations of soil physical and chemical properties under mulching treatment.

## 5 Conclusions and future research directions

Soil nutrients, enzymes, and microorganisms form a complex, interconnected system where each element influences the others, developing a dynamic and resilient soil ecosystem. This interplay is crucial for nutrient cycling, plant health, and soil quality. Long-term mulching cultivation in a “Wanzhou Red Mandarin” citrus orchard can significantly impact soil quality, microbial community structure, and fruit quality. While it can enhance soil moisture retention, potentially improve some nutrient levels, and potentially increase fruit yield, it can also alter the soil microbial community, potentially impacting its long-term health and functionality. Furthermore, it has driven a shift in microbial community composition toward more beneficial types and substantially raised the proportion of eutrophic-type microbial communities. This study offers new references and insights for constructing and utilizing healthy soil microenvironments at varying depths in orange orchards. Simultaneously, it provides fundamental data to support the rational selection of cover grass species. Further research should focus on understanding these complex interactions to optimize mulching practices for sustainable citrus production in the years to come.

## Data Availability

The original contributions presented in the study are publicly available. This data can be found at: NCBI (https://www.ncbi.nlm.nih.gov/), BioProject number PRJNA1304469.

## Footnotes

^1^ www.biocloud.net
